# Partial Tmem106b reduction does not correct abnormalities due to progranulin haploinsufficiency

**DOI:** 10.1186/s13024-018-0264-6

**Published:** 2018-06-22

**Authors:** Andrew E. Arrant, Alexandra M. Nicholson, Xiaolai Zhou, Rosa Rademakers, Erik D. Roberson

**Affiliations:** 1 Center for Neurodegeneration and Experimental Therapeutics, Alzheimer’s Disease Center, Evelyn F. McKnight Brain Institute, Departments of Neurology and Neurobiology, University of Alabama at Birmingham, 1825 University Blvd., SHEL, Birmingham, AL 1110 USA; 2 Department of Neuroscience, Mayo Clinic Jacksonville, 4500 San Pablo Road, Jacksonville, FL USA

**Keywords:** Progranulin, TMEM106B, Frontotemporal dementia, Lysosome

## Abstract

**Background:**

Loss of function mutations in progranulin (*GRN*) are a major cause of frontotemporal dementia (FTD). Progranulin is a secreted glycoprotein that localizes to lysosomes and is critical for proper lysosomal function. Heterozygous *GRN* mutation carriers develop FTD with TDP-43 pathology and exhibit signs of lysosomal dysfunction in the brain, with increased levels of lysosomal proteins and lipofuscin accumulation. Homozygous *GRN* mutation carriers develop neuronal ceroid lipofuscinosis (NCL), an earlier-onset lysosomal storage disorder caused by severe lysosomal dysfunction. Multiple genome-wide association studies have shown that risk of FTD in *GRN* mutation carriers is modified by polymorphisms in *TMEM106B*, which encodes a lysosomal membrane protein. Risk alleles of *TMEM106B* may increase TMEM106B levels through a variety of mechanisms. Brains from FTD patients with *GRN* mutations exhibit increased TMEM106B expression, and protective *TMEM106B* polymorphisms are associated with decreased TMEM106B expression. Together, these data raise the possibility that reduction of TMEM106B levels may protect against the pathogenic effects of progranulin haploinsufficiency.

**Methods:**

We crossed *Tmem106b*^*+/−*^ mice with *Grn*^*+/−*^ mice, which model the progranulin haploinsufficiency of *GRN* mutation carriers and develop age-dependent social deficits and lysosomal abnormalities in the brain. We tested whether partial Tmem106b reduction could normalize the social deficits and lysosomal abnormalities of *Grn*^*+/−*^ mice.

**Results:**

Partial reduction of Tmem106b levels did not correct the social deficits of *Grn*^*+/−*^ mice. Tmem106b reduction also failed to normalize most lysosomal abnormalities of *Grn*^*+/−*^ mice, except for β-glucuronidase activity, which was suppressed by Tmem106b reduction and increased by progranulin insufficiency.

**Conclusions:**

These data do not support the hypothesis that Tmem106b reduction protects against the pathogenic effects of progranulin haploinsufficiency, but do show that Tmem106b reduction normalizes some lysosomal phenotypes in *Grn*^*+/−*^ mice.

**Electronic supplementary material:**

The online version of this article (10.1186/s13024-018-0264-6) contains supplementary material, which is available to authorized users.

## Background

Progranulin (GRN) is a secreted glycoprotein that acts as a growth factor for many cell types, modulates inflammation, and is critical for proper lysosomal function [1–5]. Loss-of-function *GRN* mutations are one of the most common genetic causes of frontotemporal dementia (FTD), causing as much as 5–10% of all FTD cases and 20% of familial FTD cases [6–8]. *GRN* mutations are an autosomal dominant cause of FTD, and most known disease-causing mutations lead to haploinsufficiency of progranulin, with *GRN* carriers typically having less than 50% of normal circulating progranulin levels [6–9]. Progranulin haploinsufficiency is therefore thought to cause FTD in *GRN* mutation carriers. *GRN* mutations were thought to be highly penetrant until the identification of *TMEM106B* variants as an important modifier of disease risk in *GRN* carriers [10–13].

*TMEM106B* was first identified as a genetic risk factor for FTD with TDP-43 pathology (FTD-TDP) when a genome-wide association study found three single nucleotide polymorphisms (SNPs) associated with FTD-TDP risk in the region of chromosome 7 that contains *TMEM106B* [10]. Subsequent analyses identified these SNPs to be more strongly associated with FTD risk in individuals with *GRN* mutations [10], an observation that was replicated in several additional studies [11–13]. Since this initial discovery, *TMEM106B* SNPs were also found to modify disease risk in patients carrying FTD-causing *C9ORF72* repeat expansions; however, the strongest association of *TMEM106B* SNPs with FTD risk has been repeatedly found in *GRN* mutation carriers [10–16]. Thus, many genetic studies show a clear relationship of *TMEM106B* and *GRN* in FTD pathogenesis.

The mechanism by which *TMEM106B* SNPs may modify FTD risk in *GRN* carriers is unclear, though current data suggest that protective SNPs may reduce *TMEM106B* mRNA expression or protein levels. The protective allele of the SNP with the strongest FTD association, rs1990622, may be associated with lower *TMEM106B* expression [10, 17], though this has not been replicated by some other studies [11, 13, 18]. Evaluation of the *TMEM106B* SNP rs1990620, which is in complete linkage disequilibrium with rs1990622, revealed that the protective allele of rs1990620 facilitates binding of the transcriptional repressor CCCTC binding factor to *TMEM106B*, resulting in lower *TMEM106B* expression [17]. Independent analysis of a coding SNP (rs3173615 or p.T185S) in complete linkage disequilibrium with rs1990622 revealed that the protective (S) allele results in faster degradation of TMEM106B protein [19]. As a result, protein levels of the risk (T) *TMEM106B* allele were approximately twice as high as those of the protective (S) allele when expressed in a cell line, despite similar *TMEM106B* mRNA levels [19]. Perhaps consistent with a protective role of lower TMEM106B levels, increased levels of TMEM106B mRNA and protein have been observed in brains from FTD-TDP patients, both with and without *GRN* mutations [10, 20, 21]. Together, these studies form the basis of the hypothesis that reduction of TMEM106B levels may protect against the pathogenic effects of progranulin haploinsufficiency.

Reduction of TMEM106B levels might protect *GRN* mutation carriers against FTD by improving lysosomal function. Lysosomal dysfunction may be key to FTD-*GRN* pathogenesis, as progranulin is critical for proper lysosomal function. Individuals homozygous for loss of function *GRN* mutations that cause nearly complete progranulin deficiency develop the lysosomal storage disorder, neuronal ceroid lipofuscinosis [22, 23]. FTD patients with *GRN* mutations also exhibit signs of lysosomal dysfunction, such as increased levels of lysosomal proteins and lipofuscin accumulation in the brain and retina [21, 24, 25]. TMEM106B is a lysosomal membrane protein, and TMEM106B overexpression in cell culture has detrimental effects on lysosomal function, causing lysosomal enlargement, impaired acidification, reduced motility, and clustering of lysosomes in the cell body of neurons [17, 20, 26–28]. TMEM106B overexpression may increase progranulin levels, though this is likely due to lysosomal dysfunction, as lysosome-disrupting compounds also increase progranulin levels [20, 27, 29]. Although TMEM106B knockdown has been shown to affect dendritic lysosomal trafficking, lowering TMEM106B levels reduces lysosomal size and number [28, 30]. Taken together, these cell biology studies of TMEM106B indicate that reduction of TMEM106B could ameliorate the lysosomal dysfunction of *GRN* mutation carriers.

The available genetic and cell biological data indicate that reducing TMEM106B levels may have beneficial effects in *GRN* mutation carriers. In this study, we tested this hypothesis using *Grn*^*+/−*^ mice. *Grn*^*+/−*^ mice model the progranulin haploinsufficiency that causes FTD in *GRN* mutation carriers, and develop age-dependent social behavior deficits and lysosomal dysfunction in the brain [31–33]. Recent studies have tested the effects of Tmem106b overexpression or deletion in *Grn*^*−/−*^ mice, which develop severe lysosomal dysfunction and lipofuscinosis that may model the pathology of NCL in homozygous *GRN* mutation carriers. Consistent with prior cell biology studies, overexpression of human *TMEM106B* worsened the lipofuscinosis and lysosomal abnormalities of *Grn*^*−/−*^ mice [34]. In contrast, knockout of *Tmem106b* in mice suppresses expression of many lysosomal enzymes, which opposes the increases in lysosomal enzyme expression caused by progranulin deficiency [35]. As a result, crossing *Grn*^*−/−*^ mice with *Tmem106b*^*−/−*^ mice normalized the activity of some lysosomal enzymes in *Grn*^*−/−*^ mouse brain, as well as open field and elevated plus maze behavior, though it failed to normalize lipofuscinosis and gliosis [35]. However, *TMEM106B* polymorphisms only partially reduce TMEM106B expression [19], so in this study, we crossed *Tmem106b*^*+/−*^ mice with *Grn*^*+/−*^ mice to test whether partial reduction of Tmem106b would correct the behavioral deficits and lysosomal dysfunction of *Grn*^*+/−*^ mice, which model the progranulin haploinsufficiency that causes FTD-*GRN* [31–33].

## Methods

### *Generation of Grn*^*+/−*^*:Tmem106b*^*+/−*^ mice

*Tmem106b*^*+/−*^ mice were generated with a previously described gene trap vector developed by the Wellcome Trust Sanger Institute [35] and bred onto a C57BL/6 N background. *Grn*^*+/−*^*:Tmem106b*^*+/−*^ mice were generated by crossing *Tmem106b*^*+/−*^ mice with *Grn*^*+/−*^ mice on a C57BL/6 J background that were generated as previously described [31, 36]. The F1 generation was used for this study. This pairing resulted in four possible genotypes: *Grn*^*+/+*^*:Tmem106b*^*+/+*^*, Grn*^*+/+*^*:Tmem106b*^*+/−*^*, Grn*^*+/−*^*:Tmem106b*^*+/+*^*,* and *Grn*^*+/−*^*:Tmem106b*^*+/−*^. Male and female littermates from these pairings were used for all studies, with *Grn*^*+/+*^*:Tmem106b*^*+/+*^ mice serving as the control group*.* Mice were housed in the University of Alabama at Birmingham (UAB) mouse housing facility accredited by the Association for Advancement and Accreditation of Laboratory Animal Care International. The mice were maintained on a 12:12 h light/dark schedule, with the lights on at 6:00 AM and off at 6:00 PM. All experiments were conducted during the light phase under ambient room lighting. Mice were given free access to food (Harlan, #7917) and water throughout the experiment. All experiments were approved by the UAB Institutional Animal Care and Use Committee.

### Tube test for social dominance

The tube test for social dominance was conducted on both male and female mice as previously described [32], with mice of the same sex but different genotype placed in opposite ends of a 30.5 cm-long clear plastic tube. The mice were released, and the first mouse to have two feet out of the tube was considered to have lost the match. Mice were tested at age 12–14 months, an age at which *Grn*^*+/−*^ mice exhibit a stable low-dominance phenotype [32]. To confirm the presence of the expected *Grn*^*+/−*^ phenotype, we paired *Grn*^*+/+*^:*Tmem106b*^*+/+*^ mice with *Grn*^*+/−*^:*Tmem106b*^*+/+*^ mice. To test the effect of Tmem106b reduction on the *Grn*^*+/−*^ phenotype, we paired *Grn*^*+/+*^:*Tmem106b*^*+/+*^ mice with *Grn*^*+/−*^:*Tmem106b*^*+/−*^ mice. In both tests, each mouse was paired against three other mice of the opposing genotype over three rounds of testing, as previously described [32]. Matches from each test (*Grn*^*+/+*^:*Tmem106b*^*+/+*^ vs. *Grn*^*+/−*^:*Tmem106b*^*+/+*^ or *Grn*^*+/−*^:*Tmem106b*^*+/−*^) were interspersed to avoid any confounding effects of test order. For example a *Grn*^*+/+*^:*Tmem106b*^*+/+*^ mouse would face a *Grn*^*+/−*^:*Tmem106b*^*+/+*^ mouse in round 1 of testing, then a *Grn*^*+/−*^:*Tmem106b*^*+/−*^ mouse in round 2, with the genotype of the opponent alternating over a total of six rounds of testing. We did not observe an effect of testing round on the dominance phenotypes, so the repeated testing did not measurably affect the behavioral performance of the mice, as we have previously observed [32]. On a separate day, we paired *Grn*^*+/−*^:*Tmem106b*^*+/+*^ mice against *Grn*^*+/−*^:*Tmem106b*^*+/−*^ mice, and *Grn*^*+/+*^:*Tmem106b*^*+/+*^ mice against *Grn*^*+/+*^:*Tmem106b*^*+/−*^ mice to assess the effect of Tmem106b reduction on social dominance within *Grn*^*+/−*^ mice. This test was also conducted over three rounds of testing.

### Western blot

The following antibodies were used for western blot: LAMP-1 (1:250, Developmental Studies Hybridoma Bank #1D4B), Tmem106b (1:500, Bethyl Laboratories, #A303-439A), progranulin (1:500, rabbit anti-progranulin polyclonal antibody [37]), HexA (1:500, Abcam, #189685), GCase (1:500, Santa Cruz, #sc-166,407), GAPDH (1:5000, MilliporeSigma #MAB374). All primary antibody incubations were conducted overnight at 4 °C.

Frontal cortex samples were homogenized in lysis buffer (50 mM Tris, 150 mM NaCl, 5 mM EDTA, 1% Triton X-100, 0.1% sodium deoxycholate) and centrifuged at 5000 x *g* for 10 min at 4 °C. Protein concentration of the lysates was assessed by Bradford assay (Coomassie Plus, Thermo Scientific). For western blot, samples were diluted with 4X LDS sample buffer (ThermoFisher Scientific) and 10X Bolt sample reducing agent (ThermoFisher Scientific) and heated for 10 min at 70 °C. 20 μg of protein was loaded per lane on 4–12% bis-tris gels. After electrophoresis, proteins were transferred to Immobilon-FL PVDF membranes (MilliporeSigma) and blocked with 50% Odyssey blocking buffer (LI-COR Biotechnologies) prior to overnight incubation with primary antibody. Antibody labeling was detected using an IR-dye-conjugated species-matched secondary antibody (LI-COR Biotechnologies).

Western blot of Tmem106b in brain tissue proved to be incompatible with bis-tris polyacrylamide gels. For these blots, 20–30 μg of protein was loaded onto 12% TGX gels (Biorad) and transferred onto Immobilon-FL PVDF membranes (MilliporeSigma) in Dunn carbonate buffer [38]. The membranes were blocked in 5% milk prior to probing with antibodies as described above. All blots were scanned on an Odyssey scanner (LI-COR Biotechnologies) and quantitated with Image Studio Lite software (LI-COR Biotechnologies).

### Enzyme activity assays

Fluorogenic assays were used to determine the enzymatic activity of β-hexosaminidase A (HexA), β-glucocerebrosidase (GCase), and β-glucuronidase (Gusb) in frontal cortical lysates. Frontal cortex samples were prepared without protease inhibitors as described for western blot. HexA and Gusb activity were determined by incubating frontal cortical lysates (5 μg protein per well) with 2 mM fluoregenic substrates for HexA (4-methylumbelliferyl-2-acetamido-2-deoxy-6-sulfate-β-D-glucopyranoside, Research Products International) or Gusb (4-methylumbelliferyl-β-D-glucuronide hydrate, MilliporeSigma) in 10 mM sodium citrate buffer, pH 4.2 [39]. The reactions proceeded for 1 h at 37 °C and stopped by addition of 0.2 M glycine, 0.2 M sodium carbonate. GCase activity was determined by incubating frontal cortical lysates (10 μg protein per well) with 1 mM fluorogenic GCase substrate (4-Methylumbelliferyl β-D-glucopyranoside, MilliporeSigma) in citrate-phosphate buffer, pH 4.6 containing final concentrations of 1% bovine serum albumin, 0.25% triton X-100, 0.25% taurocholic acid, and 1 mM EDTA [40]. The reactions proceeded for 1 h at 37 °C and stopped by addition of 0.4 M glycine, pH 10.8. Specific GCase activity was confirmed for each sample by subtracting the activity of wells containing 0.18 mM conduritol β-epoxide (Enzo Life Sciences), a GCase inhibitor. All assays were carried out in white, opaque 96-well plates and read on a Synergy 2 plate reader (Biotek Instruments) with an excitation wavelength of 360 nm and an emission wavelength of 440 nm. The amount of methylumbelliferone freed during the reaction was quantitated relative to a standard curve of methylumbelliferone (4-MU) run on each plate, and results were calculated as nmol 4-MU/hour/mg protein.

### PNGase digestion

Frontal cortical lysates prepared as described above were incubated overnight at 37 °C with PNGase F (New England Biolabs) according the manufacturer’s instructions. 50 μg of protein were included in each reaction. For each sample, a control tube was incubated without PNGase F enzyme. After incubation, the samples were diluted with 4X LDS sample buffer (ThermoFisher Scientific) and 10X Bolt sample reducing agent (ThermoFisher Scientific) and heated for 10 min at 70 °C before analysis by western blot as described above.

### Statistics

**Fig. 1:**
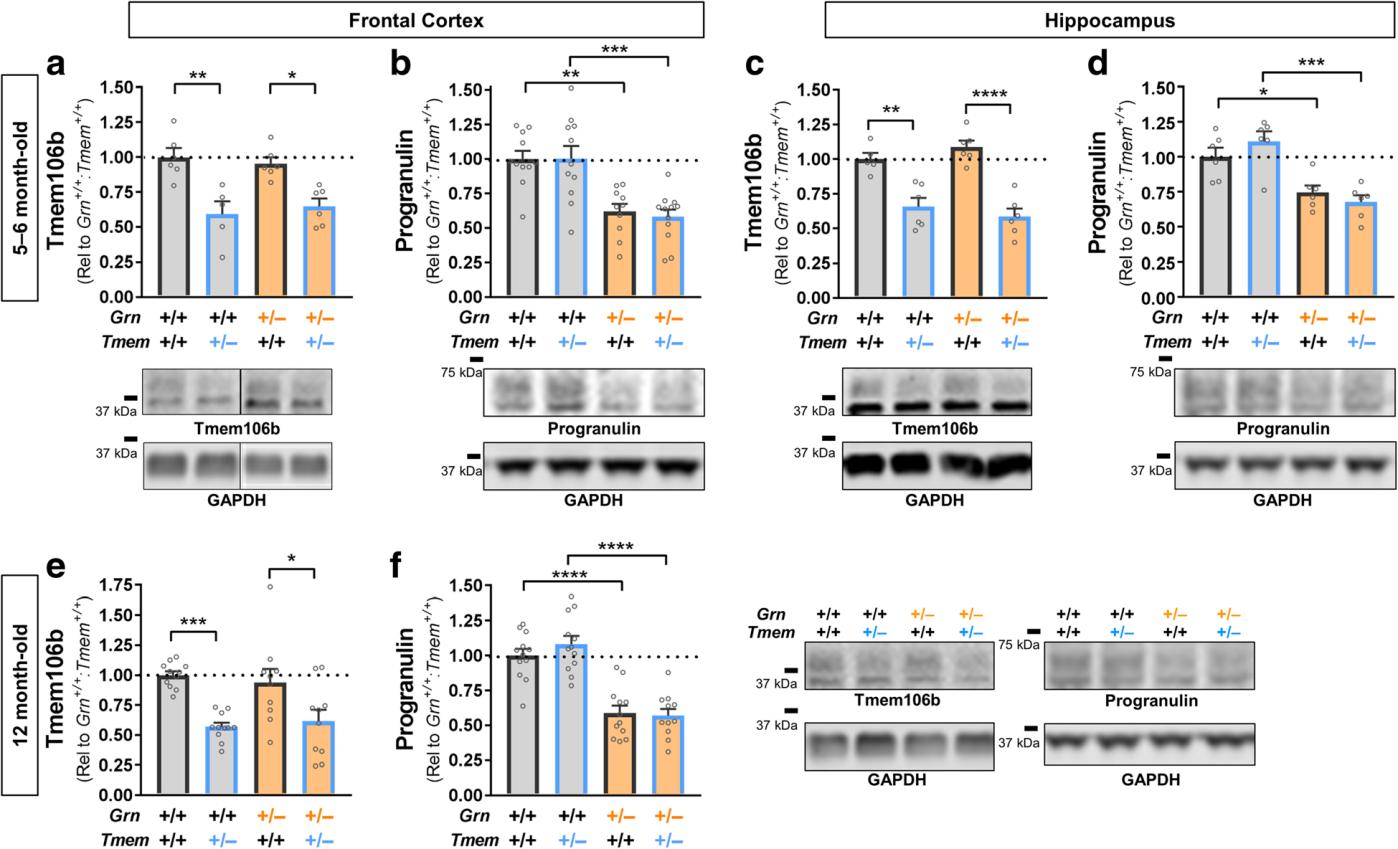
*Grn*^*+/−*^:*Tmem106b*^*+/−*^ Exhibit a 40–50% Reduction in Both Progranulin and Tmem106b Protein Levels. In the frontal cortex of 5–6 month-old mice, the knockout *Grn* and *Tmem106b* alleles mediated the expected reduction of Tmem106b (**a,** ANOVA effect of *Tmem106b*, *p* < 0.0001) and progranulin (**b**, ANOVA effect of *Grn*, *p* < 0.0001) protein levels. Similar results were obtained in the hippocampus, with the expected reduction of Tmem106b (**c**, ANOVA effect of *Tmem106b*, *p* < 0.0001) and progranulin (**d**, ANOVA effect of *Grn*, *p* < 0.0001). Tmem106b (**e**, ANOVA effect of *Tmem106b*, *p* < 0.0001) and progranulin (**f**, ANOVA effect of *Grn*, *p* < 0.0001) were also reduced in the frontal cortex of 12 month-old mice. In all cases, the knockout *Grn* and *Tmem106b* alleles reduced their target protein levels by ~ 35–45% regardless of the genotype of the other allele. * = *p* < 0.05, ** = *p* < 0.01, *** = *p* < 0.001, **** = *p* < 0.0001 by Tukey’s post-hoc test. *n* = 5–12 mice per group for 5- to 6-month-old mice and 10–11 per group for 12-month-old mice

**Fig. 2:**
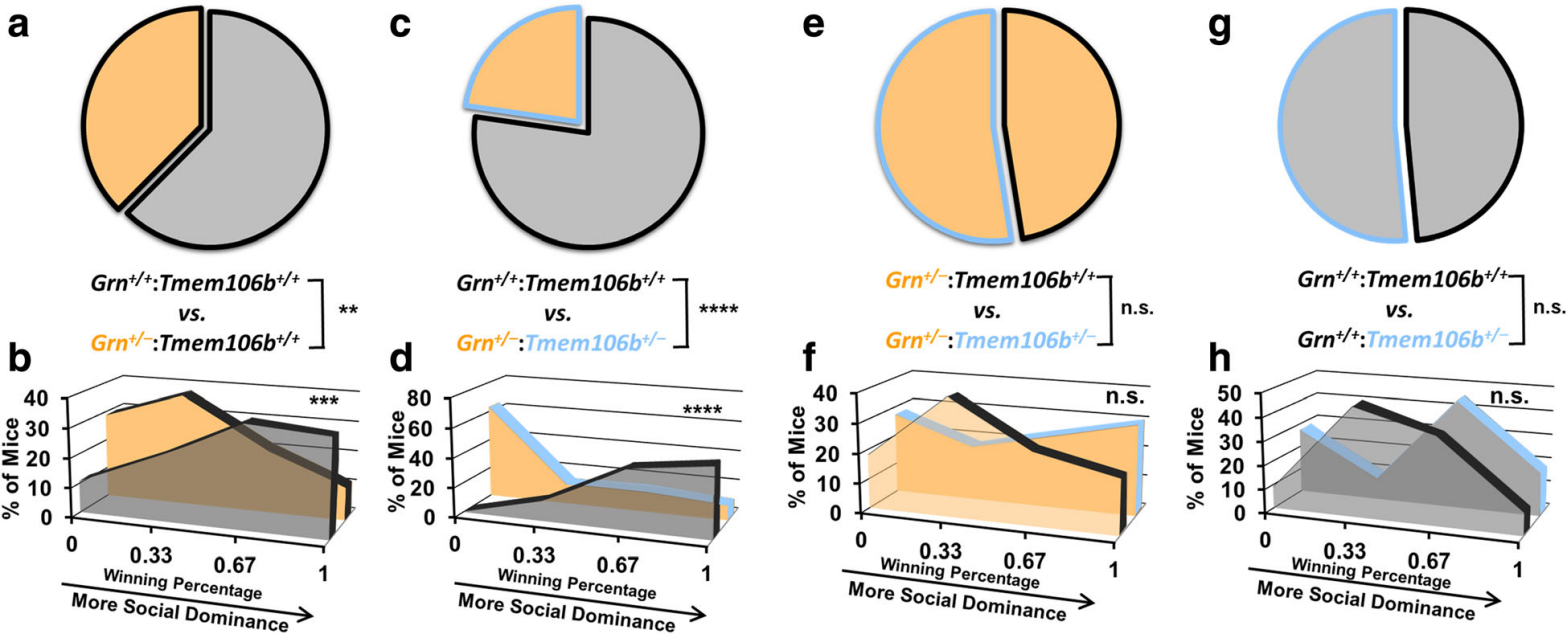
Tmem106b Reduction Does Not Rescue the Social Dominance Deficits of *Grn*^*+/−*^ Mice. When paired against *Grn*^*+/+*^:*Tmem*^*+/+*^ mice, *Grn*^*+/−*^:*Tmem*^*+/+*^ mice exhibited the expected losing phenotype as shown by both wins per genotype (**a**, Binomial test, *p* = 0.0053, *n* = 133 matches) and winning percentage (**b**, Mann-Whitney test, *p* = 0.0016, *n* = 45–46 mice per genotype). *Grn*^*+/−*^:*Tmem*^*+/−*^ mice also exhibited a losing phenotype versus *Grn*^*+/+*^:*Tmem*^*+/+*^ mice (**c**, Binomial test, *p* < 0.0001, **d**, Mann-Whitney test, *p* < 0.0001, *n* = 30 mice per genotype). Tmem106b reduction did not i change social dominance between either *Grn*^*+/−*^ mice(**h**, Binomial test, *p* = 0.6879, **i**, Mann-Whitney test, *p* = 0.6198, *n* = 33 mice per genotype) or *Grn*^*+/+*^ mice (**f**, Binomial test, *p* > 0.9999 **g**, Mann-Whitney test, *p* = 0.6914, *n* = 11 mice per genotype)

**Fig. 3:**
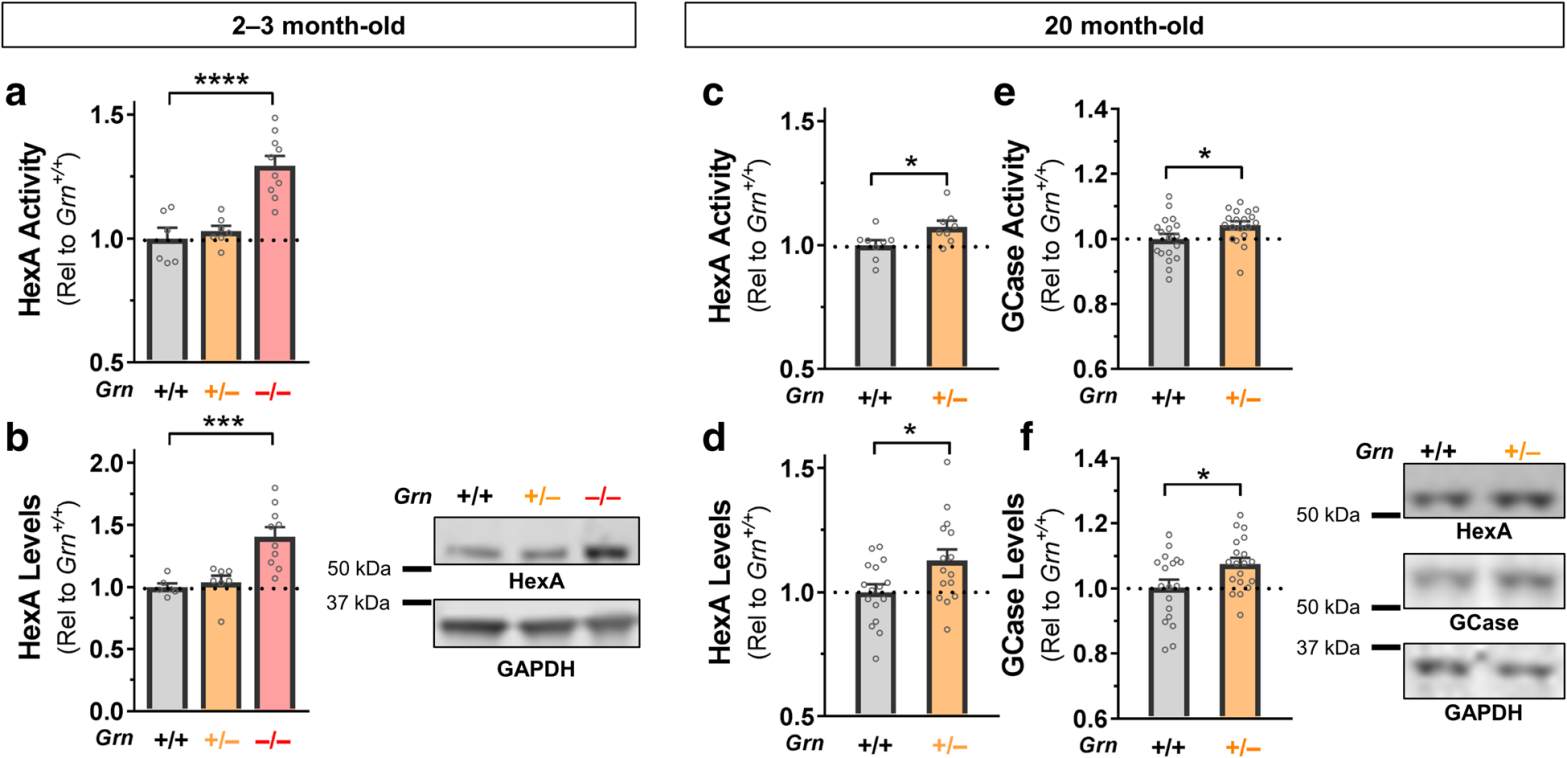
Increased Enzyme Activity is Associated with Increased Enzyme Levels in *Grn*^*+/−*^ and *Grn*^*−/−*^ Mice. *Grn*^*−/−*^ mice exhibited elevated HexA activity in the frontal cortex at age 2–3 months (**a**, ANOVA effect of *Grn*, *p* < 0.0001, Dunnett’s post-hoc test, *p* = 0.0001), which was associated with reduced HexA protein levels (**b**, ANOVA effect of *Grn*, *p* = 0.0003, Dunnett’s post-hoc test, *p* = 0.0007). While *Grn*^*+/−*^ mice did not exhibit elevated HexA activity at 2–3 months of age, 20 month-old *Grn*^*+/−*^ mice exhibited elevated HexA (**a**, *t* test, *p* = 0.037) and GCase (**c**, *t* test, *p* = 0.0408) activity in the frontal cortex. Consistent with this increased activity, *Grn*^*+/−*^ mice exhibited elevated HexA (**b**, *t* test, *p* = 0.0262) and GCase (**d**, *t* test, *p* = 0.0222) protein levels. * = *p* < 0.05, *** = *p* < 0.001, **** = *p* < 0.0001. *n* = 8–19 mice per genotype

**Fig. 4:**
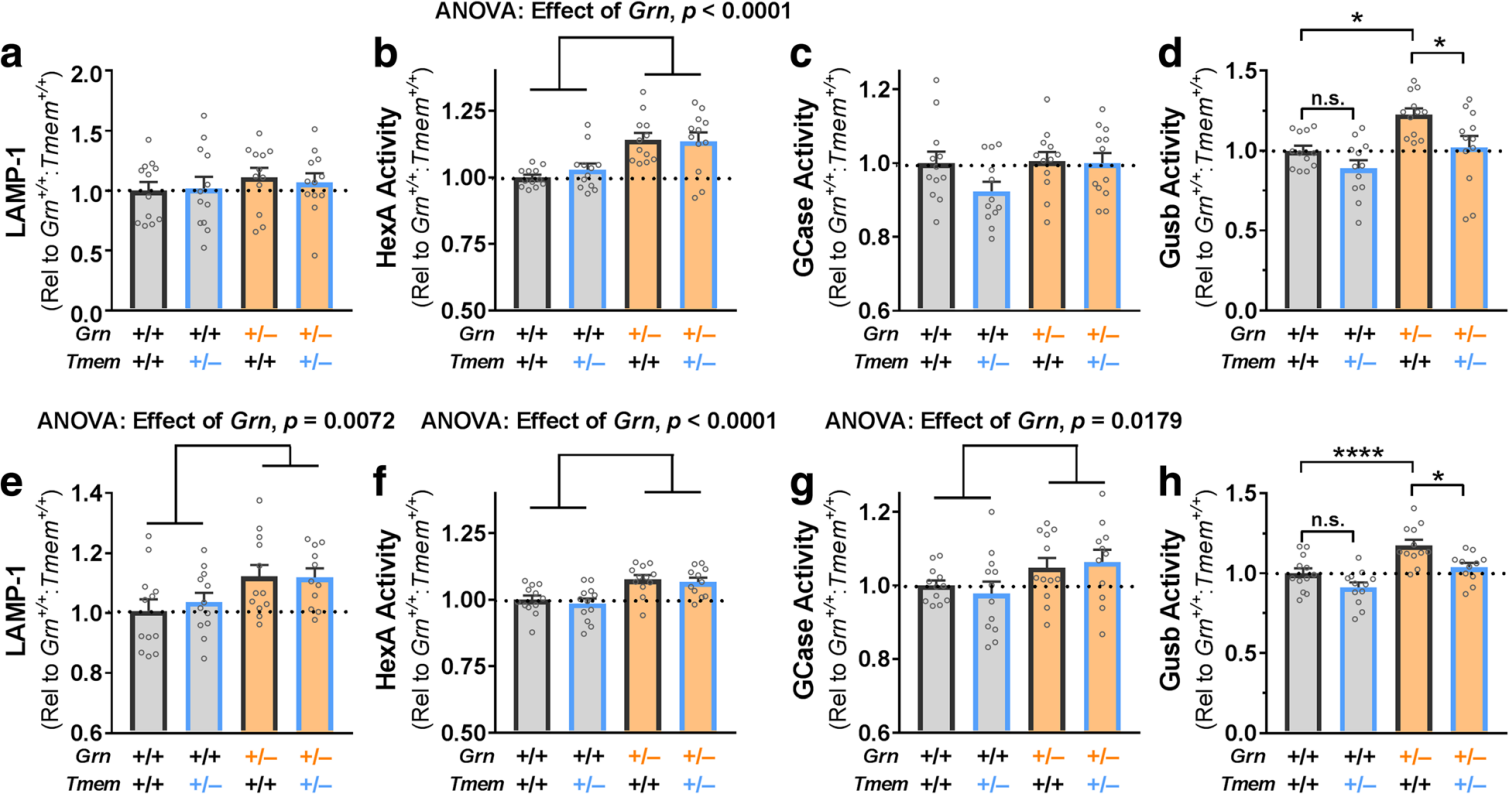
Tmem106b Reduction Does Not Rescue Most Lysosomal Phenotypes of *Grn*^*+/−*^ Mice. Frontal cortex samples from 5- to 6-month-old *Grn*^*+/−*^ mice exhibited no differences from wild-type in LAMP-1 levels (**a**) or GCase activity (**c**), but increased HexA activity (**b**, **** = ANOVA effect of *Grn*, *p* < 0.0001) and Gusb activity (**d**, ANOVA effect of *Grn*, *p* = 0.0009). Tmem106b reduction had no significant effect on LAMP-1, HexA, or GCase in 5- to 6-month-old mice, but significantly reduced Gusb activity (ANOVA effect of *Tmem106b*, *p* = 0.0031, * = *p* < 0.05 by Tukey’s post-hoc test). Frontal cortex samples from 12-month-old *Grn*^*+/−*^ mice exhibited increased LAMP-1 levels (**e**, ** = ANOVA effect of *Grn*, *p* = 0.0072), HexA activity (**f**, **** = ANOVA effect of *Grn*, *p* < 0.0001), GCase activity (**g**, * = ANOVA effect of *Grn*, *p* = 0.0179), and Gusb activity (**h**, ANOVA effect of *Grn*, *p* < 0.0001). As in the younger mice, Tmem106b reduction had no significant effect on LAMP-1, HexA, or GCase in 12 month-old mice, but significantly reduced Gusb activity (ANOVA effect of *Tmem106b*, *p* = 0.0010, * = *p* < 0.05, **** = *p* < 0.0001 by Tukey’s post-hoc test). *n* = 12 mice per group for 5- to 6-month-old mice and 11–12 mice per group for 12-month-old mice

**Fig. 5:**
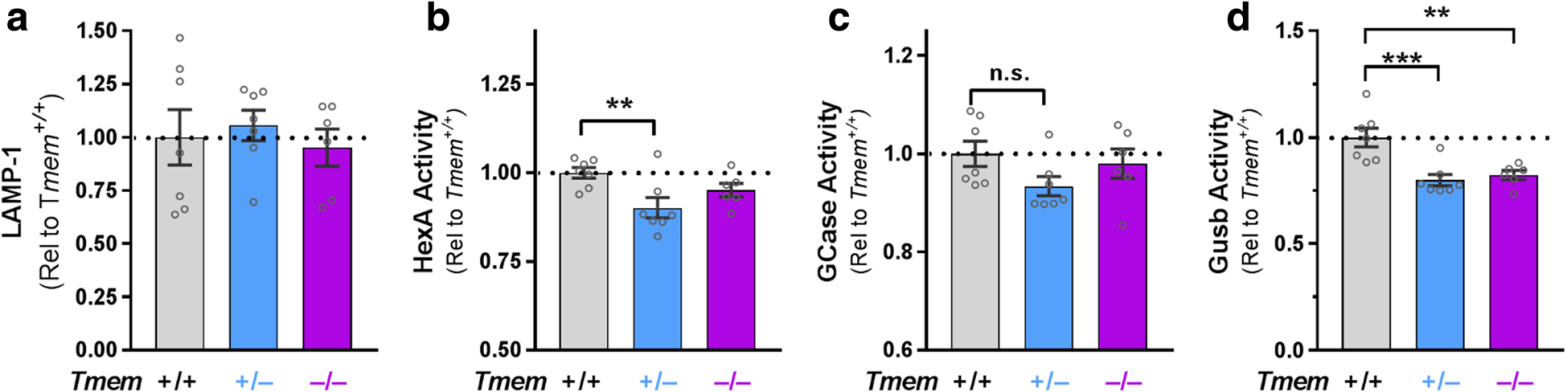
Tmem106b Reduction Suppresses Activity of β-Glucuronidase. Tmem106b reduction had no significant effect on LAMP-1 levels (**a**) or GCase activity (**c**) in the frontal cortex of 2- to 5-month-old mice. HexA activity was significantly reduced in *Tmem106b*^*+/−*^ mice (**b**, ANOVA effect of *Tmem106b*, *p* = 0.0175, ** = *p* = 0.0095 by Dunnett’s post-hoc test). Gusb activity was significantly reduced in both *Tmem106b*^*+/−*^ and *Tmem106b*^*−/−*^ mice (**d**, ANOVA effect of *Tmem106b*, *p* = 0.0007, ** = *p* < 0.01, *** = *p* < 0.001 by Dunnett’s post-hoc test). *n* = 6–7 mice per genotype

Tmem106b, progranulin, and LAMP-1 levels, as well as HexA, GCase, and Gusb activity in *Grn*^*+/−*^:*Tmem106b*^*+/−*^ mice (Figs. 1 and 4), were analyzed by two-way ANOVA with factors of *Tmem106b* and *Grn*. Significant main effects or interactions were followed by Tukey’s post-hoc test. HexA and GCase activity and levels from aged *Grn*^*+/−*^ mice (Fig. 3c–f) were analyzed by *t* test. LAMP-1 levels and HexA, GCase, and Gusb activity in *Tmem106b*^*−/−*^ mice (Fig. 5), as well as HexA activity and levels for young mice (Fig. 3a, b) were analyzed by one-way ANOVA, and a significant genotype effect was followed by Dunnett’s post-hoc test to compare heterozygous and homozygous knockout mice to wild-type. The number of wins per group in the tube test (Fig. 2a, c, e, g) was analyzed by binomial test to compare the observed vs. expected outcome, with the expected outcome set at 50% wins per group. The winning percentage in the tube test (Fig. 2b, d, f, h) was analyzed by Mann-Whitney test. Two-tailed *p* values were calculated for all analyses, with α set at 0.05. All analyses were conducted with GraphPad Prism 7 (GraphPad Software).

## Results

### Grn^+/−^:Tmem106b^+/−^ mice exhibit a similar reduction in cortical and hippocampal progranulin and Tmem106b protein levels

After generating *Grn*^*+/−*^:*Tmem106b*^*+/−*^ mice, we measured progranulin and Tmem106b levels in the frontal cortex and hippocampus of 5- to 6-month-old mice. We observed a similar reduction of Tmem106b levels (~ 35–45%) in *Tmem106b*^*+/−*^ mice regardless of *Grn* genotype (Fig. 1a, c), and a similar reduction in progranulin levels (~ 35–45%) in *Grn*^*+/−*^ mice regardless of *Tmem106b* genotype (Fig. 1b, d). Similarly, the 12-month-old mice used for behavioral studies described below exhibited reduction of progranulin and Tmem106b without signs of interaction between the two knockout alleles (Fig. 1e, f). We observed no effect of *Tmem106b* genotype on progranulin levels, and no effect of *Grn* genotype on Tmem106b levels.

### Tmem106b reduction does not rescue the low social dominance phenotype of Grn^+/−^ mice

To test the hypothesis that Tmem106b reduction may rescue deficits induced by progranulin haploinsufficiency, we tested wild-type (*Grn*^+/+^:*Tmem106b*^+/+^), *Grn*^*+/−*^:*Tmem106b*^+/+^, and *Grn*^*+/−*^:*Tmem106b*^*+/−*^ mice in the tube test for social dominance at age 11–12 months. *Grn*^*+/−*^ mice develop a stable low dominance phenotype in this task after 9 months of age, making it a useful behavioral screen for social deficits induced by progranulin insufficiency [32]. When tested against wild-type mice, *Grn*^*+/−*^:*Tmem106b*^*+/+*^ mice exhibited a low dominance phenotype of a similar magnitude as previously reported (Fig. 2a, b) [32]. Tmem106b reduction did not correct this phenotype, as *Grn*^*+/−*^:*Tmem106b*^*+/−*^ mice also exhibited a low dominance phenotype against wild-type mice (Fig. 2c, d). If anything, it appeared that the low dominance phenotype of *Grn*^*+/−*^:*Tmem106b*^*+/−*^ mice might have even been worse than that of *Grn*^*+/−*^:*Tmem106b*^*+/+*^ mice. To determine if this was a significant effect, i.e. whether Tmem106b reduction significantly reduced dominance in *Grn*^*+/−*^ mice, we directly compared dominance in *Grn*^*+/−*^:*Tmem106b*^*+/+*^ mice and *Grn*^*+/−*^:*Tmem106b*^*+/−*^ mice by pairing the groups against each other in the tube test. We observed no effect of *Tmem106b* genotype in this comparison (Fig. 2e, f). We similarly saw no effect of *Tmem106b* genotype on social dominance in *Grn*^*+/+*^ mice (Fig. 2g, h). Based on these data, we conclude that Tmem106b reduction does not rescue the low dominance phenotype of *Grn*^*+/−*^ mice, but does not worsen it either.

### Grn^+/−^ mice exhibit elevated levels and activity of lysosomal enzymes in the frontal cortex

We next asked if *Tmem106b* reduction had any effect on lysosomal abnormalities in *Grn*^*+/−*^ mice. We previously reported that *Grn*^*+/−*^ and *Grn*^*−/−*^ mice have increased levels of LAMP-1 in the frontal cortex at around 12 months of age [33]. To gain a more complete picture of the lysosomal abnormalities caused by progranulin insufficiency, we measured activity of the lysosomal enzyme *β-*hexosaminidase A (HexA), which has been reported to have increased expression and activity in *Grn*^*−/−*^ mice [35, 41]. Consistent with these prior reports, we observed elevated HexA activity and protein levels in the frontal cortex of *Grn*^*−/−*^ mice aged 2–3 months (Fig. 3a, b). *Grn*^*+/−*^ mice did not have significantly elevated HexA at this age, though it is notable that they also lack detectable behavioral phenotypes at 2–3 months [32]. To determine if *Grn*^*+/−*^ mice would exhibit lysosomal enzyme abnormalities at a more advanced age, we analyzed HexA activity and protein levels in the frontal cortex of 20-month-old wild-type and *Grn*^*+/−*^ mice. These 20-month-old *Grn*^*+/−*^ mice exhibited elevated HexA activity (Fig. 3c) and protein levels (Fig. 3d). To determine if similar changes could be observed in other lysosomal enzymes, we measured activity and protein levels of *β-*glucocerebrosidase (GCase), which is reported to be improperly trafficked in *Grn*^*−/−*^ mice [42]. Similar to HexA, 20-month-old *Grn*^*+/−*^ mice exhibited elevated GCase activity (Fig. 3e) and protein levels (Fig. 3f). Both HexA and GCase are glycosylated to facilitate their maturation and trafficking to the lysosome, and our western blots measured levels of the fully glycosylated proteins (Additional file 1: Figure S1). Along with the previously reported elevations in LAMP-1 levels [33], these novel lysosomal abnormalities show that brains from *Grn*^*+/−*^ mice have elevated levels of lysosomal membrane proteins and mature lysosomal enzymes, which may be consistent with a compensatory response to an underlying lysosomal dysfunction.

### Tmem106b reduction does not normalize most lysosomal abnormalities of Grn^+/−^ mice

We next tested whether Tmem106b reduction could normalize the elevated levels of LAMP-1 and activity of HexA and GCase in *Grn*^*+/−*^ mice. Both HexA and GCase are involved in metabolism of glycosphingolipids [43], so we also measured activity of *β-*glucuronidase (Gusb), an enzyme involved in metabolism of glycosaminoglycans, to gain insight into a lysosomal enzyme involved in a different metabolic pathway and to provide a broader perspective on lysosomal enzyme changes in *Grn*^*+/−*^ mice. *Grn*^*+/−*^ mice begin to show behavioral deficits around 6 months of age [31], and have well-established behavioral and lysosomal abnormalities by 12 months of age [31–33], so we measured LAMP-1 levels and the activity of HexA, GCase, and Gusb in frontal cortex samples from 5- to 6-month-old and 12-month-old mice to assess lysosomal abnormalities in *Grn*^*+/−*^ mice in the early and established stages of abnormal behavior.

While LAMP-1 and GCase activity remained unchanged in *Grn*^+/−^ mice at 5- to 6-months of age (Fig. 4a, c), we observed significantly increased HexA (Fig. 4b) and Gusb (Fig. 4d) activity in the frontal cortex, showing that *Grn*^*+/−*^ mice exhibit lysosomal abnormalities around the same age that behavioral deficits begin to develop [31]. In 12-month-old *Grn*^*+/−*^ mice, we again observed increased HexA (Fig. 4f) and Gusb (Fig. 4h) activity, as well as increased LAMP-1 levels (Fig. 4e) and GCase activity (Fig. 4g). These data show that lysosomal abnormalities worsen in an age-dependent manner in *Grn*^*+/−*^ frontal cortex along a parallel time-course with the ongoing development of behavior deficits [32]. We were able to measure protein levels of HexA and GCase by western blot in 12-month-old mice, but did not observe significant increases in *Grn*^*+/−*^ mice (data not shown). The failure to detect a change in enzyme levels may be due to the less quantitative nature of western blot relative to fluorometric enzyme assay, and could also be due to ongoing increases in enzyme levels between 12 and 20 months of age in *Grn*^+/−^ mice.

Tmem106b reduction failed to normalize the increased LAMP-1 levels (Fig. 4e), HexA activity (Fig. 4b, f), and GCase activity (Fig. 4g) of *Grn*^*+/−*^ mice, leading us to conclude that Tmem106b reduction does not normalize the lysosomal dysfunction of *Grn*^*+/−*^ mice. However, Tmem106b reduction did compensate for the increased Gusb activity of *Grn*^*+/−*^ mice at both 5–6 and 12 months of age (Fig. 4d, h). Tmem106b reduction also produced a trend for lowering Gusb activity in *Grn*^*+/+*^ mice (Fig. 4d, h), potentially indicating that the normalization of Gusb activity in *Grn*^*+/−*^:*Tmem106b*^*+/−*^ mice is due to independent suppression of Gusb activity by Tmem106b reduction instead of a correction of lysosomal dysfunction in *Grn*^*+/−*^ mice, i.e. “pseudonormalization”.

### Tmem106b reduction suppresses β-Glucuronidase activity independent of progranulin genotype

To test whether suppression of Gusb activity by Tmem106b reduction, independently of progranulin, could explain the normalization of Gusb activity in *Grn*^*+/−*^:*Tmem106b*^*+/−*^ mice, we analyzed the previously described lysosomal markers in *Tmem106b*^*+/+*^, *Tmem106b*^*+/−*^, and *Tmem106b*^*−/−*^ mice on a *Grn*^*+/+*^ background. As predicted, we observed a significant suppression (~ 20%) of Gusb activity in *Tmem106b*^*+/−*^ and *Tmem106b*^*−/−*^ mice (Fig. 5d). *Tmem106b*^*+/−*^ and *Tmem106b*^*−/−*^ mice did not exhibit significant decreases in LAMP-1 activity (Fig. 5a) or in GCase activity (Fig. 5c). Interestingly, *Tmem106b*^*+/−*^, but not *Tmem106b*^*−/−*^ mice, exhibited a mild suppression (~ 10%) of HexA activity (Fig. 5b). This was somewhat unexpected, as *Grn*^*+/+*^:*Tmem106b*^*+/−*^ mice in our previous experiment did not have suppressed HexA activity (Fig. 4b, f). This discrepancy may be related to the age of the mice, as these mice were aged 2–5 months (average age around 4 months), while the experiments in Fig. 4 were done on older mice (average ages of around 6 and 12 months). HexA activity was not suppressed in *Tmem106b*^*−/−*^ mice, which has also been observed by others [35]. In previous studies with progranulin-insufficient mice, we have observed phenotypes in *Grn*^*+/−*^ mice that were not present in *Grn*^*−/−*^ mice, so these data might indicate mild, differential effects of partial Tmem106b reduction versus total deletion of Tmem106b. In summary, we conclude that Tmem106b reduction suppresses Gusb activity, but may also have a weaker, age-dependent suppressive effect on HexA activity.

## Discussion

This study shows that an approximate 50% reduction of Tmem106b levels is not sufficient to rescue behavioral and most lysosomal abnormalities in a *Grn*^*+/−*^ mouse model of FTD. Partial Tmem106b reduction had measurable effects on lysosomal function, particularly Gusb activity, but this did not correct the other lysosomal abnormalities or the loss of social dominance seen in *Grn*^*+/−*^ mice. These data suggest that the strong protective effects of certain *TMEM106B* SNPs in FTD-*GRN* may result from mechanisms apart from reduced TMEM106B levels.

This study was premised on consistently reproduced evidence that SNPs in *TMEM106B* modulate risk for FTD-TDP, with a particularly strong effect in *GRN* mutation carriers [10–16]. While the evidence that these *TMEM106B* SNPs protect against FTD in *GRN* carriers is strong, the mechanism by which they do so is not yet clear. As previously discussed, several lines of evidence indicate that protective alleles of these SNPs may reduce TMEM106B protein levels through either reduced expression of *TMEM106B* RNA or by enhanced degradation of TMEM106B protein [10, 11, 13, 17–19]. We modeled this potentially protective TMEM106B reduction using *Tmem106b*^*+/−*^ mice, which exhibited a 35–45% reduction in Tmem106b protein levels (Fig. 1a, c, f). We crossed *Tmem106b*^*+/−*^ mice with *Grn*^*+/−*^ mice to test the hypothesis that Tmem106b reduction could rescue deficits induced by progranulin insufficiency.

Tmem106b reduction failed to normalize the behavioral and most lysosomal deficits of *Grn*^*+/−*^ mice, showing that partial Tmem106b reduction does not rescue most deficits induced by progranulin insufficiency in mice. In considering the implications of these results for *TMEM106B*/*GRN* interactions in humans, it is important to note the strengths and weaknesses of the mouse models used for this study. The gene-trap method used to knock out Tmem106b in this study leaves the first three exons of the *Tmem106b* gene in place [35], raising the potential for Tmem106b fragments to persist from the knockout allele. However, we did not detect any such fragments by western blot using an antibody recognizing the amino-terminal part of the protein that should be present in any such fragments, and we observed the expected loss of nearly 50% of Tmem106b protein levels (Fig. 1). Nonetheless, the potential presence of Tmem106b fragments from the knockout allele of this *Tmem106b* mouse model raises a need for future studies with *Tmem106b* knockout models featuring complete deletion of the *Tmem106b* coding region.

We consider the partial reduction of both Tmem106b and progranulin through the use of *Tmem106b*^*+/−*^ and *Grn*^*+/−*^ mice to be a strength of this study, as the heterozygous knockouts model the partial reduction in gene expression caused by both protective *TMEM106B* SNPs and *GRN* haploinsufficiency. Protective *TMEM106B* SNPs are associated with reduced *TMEM106B* mRNA or protein levels, but not total loss of TMEM106B [10, 17, 19]. *Tmem106b*^*+/−*^ mice thus model the partial TMEM106B reduction mediated by protective SNPs, although they may not achieve the same magnitude of reduction as the *TMEM106B* SNPs. FTD-*GRN* is caused by heterozygous mutations, while individuals homozygous for pathogenic *GRN* mutations develop neuronal ceroid lipofuscinosis instead of FTD [22, 23], making *Grn*^*+/−*^ mice a closer genetic model of human FTD-*GRN* than *Grn*^*−/−*^ mice. *Grn*^*+/−*^ provide an accurate model of most of the common FTD-causing *GRN* mutations, which reduce progranulin levels through nonsense-mediated decay and cause a complete loss of protein from the mutant allele [6]. However, other *GRN* mutations may produce some dysfunctional protein product [44, 45]. It may therefore be of interest to study the effects of Tmem106b reduction in knock-in models of *GRN* mutations that produce some protein product.

**Fig. 6:**
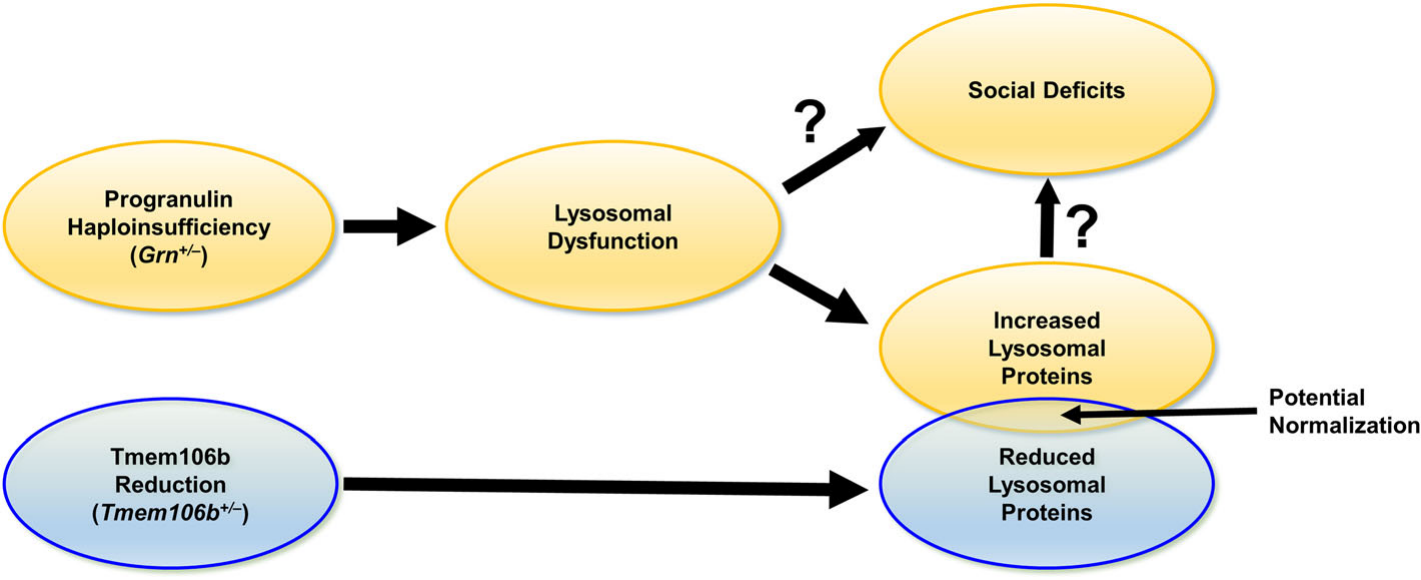
Proposed Model of the Effects of Progranulin and Tmem106b in *Grn*^*+/−*^:*Tmem106b*^*+/−*^ Mice. The current study and others [35], indicate that Tmem106b reduction in progranulin insufficient mice normalizes some lysosomal abnormalities induced by progranulin insufficiency, but fails to rescue the most abnormalities caused by progranulin insufficiency. In this model, progranulin insufficiency causes lysosomal dysfunction, which may then cause the social deficits of *Grn*^*+/−*^ mice. In addition, levels of many lysosomal proteins are increased, probably as a result of increased lysosomal gene expression via transcription factor EB. This increase in lysosomal proteins may also contribute to social behavior deficits, or may be a parallel phenomenon resulting from the underlying lysosomal dysfunction. In contrast, Tmem106b reduction suppresses the expression of many lysosomal genes (Figs. 3, 4 and [35]). Genes affected by both progranulin insufficiency and Tmem106b reduction, such as Gusb (Fig. 3d, h), may have normalized activity in *Grn*^*+/−*^:*Tmem106b*^*+/−*^ mice, but lysosomal dysfunction and social deficits remain intact in *Grn*^*+/−*^:*Tmem106b*^*+/−*^ mice

Although the lysosomal abnormalities of *Grn*^*+/−*^ mice are much milder than those of *Grn*^*−/−*^ mice, *Grn*^*+/−*^ mice have interesting, likely disease-relevant abnormalities, and this study adds to the known lysosomal changes of *Grn*^*+/−*^ mice. *Grn*^*+/−*^ mice have well-documented behavioral abnormalities [31–33], but the cellular basis for these abnormalities has remained elusive. Observation of lysosomal abnormalities in brains from FTD patients with *GRN* mutations has led to the hypothesis that progranulin haploinsufficiency impairs lysosomal function, albeit less severely than complete progranulin deficiency, and that FTD-*GRN* may be on a continuum of lysosomal dysfunction with NCL due to *GRN* mutations [21, 25]. These novel lysosomal abnormalities in *Grn*^*+/−*^ mice (Fig. 3), as well as previous reports of increased LAMP-1 levels [33] and the more dramatic lysosomal abnormalities of *Grn*^*−/−*^ mice [21, 35, 41, 46], indicate that mice may model the gene-dose effect of progranulin insufficiency on lysosomal function. These lysosomal changes may be related to the behavioral deficits of *Grn*^*+/−*^ mice, as they appear in a roughly similar time-course [32] and are normalized along with social dominance deficits by boosting progranulin levels with an AAV vector [33].The enzymes with increased activity in *Grn*^*+/−*^ mice (HexA, GCase, and Gusb) are all part of the CLEAR (coordinated lysosomal expression and regulation) network of lysosomal genes that is activated in response to lysosomal dysfunction [47, 48]. Given that the increased activity of HexA and GCase in *Grn*^+/−^ mice is associated with increased levels of these enzymes, these increases are likely to be compensatory responses to as-yet uncharacterized lysosomal deficits induced by progranulin insufficiency (Fig. 6). In this context, it is unsurprising that normalization of Gusb activity alone failed to alter the social dominance phenotype of *Grn*^*+/−*^ mice.

The counteraction of some lysosomal abnormalities in progranulin-insufficient mice by Tmem106b reduction (Fig. 4) is consistent with a recent study in which *Tmem106b*^−/−^ mice were crossed with *Grn*^*−/−*^ mice [35]. This study reported opposing effects of *Grn* and *Tmem106b* knockout on expression of many lysosomal genes, with *Grn* knockout elevating expression and *Tmem106b* knockout reducing expression [35]. For lysosomal enzymes affected by both *Grn* and *Tmem106b*, their opposing effects resulted in enzyme activity that did not differ from wild-type in *Grn*^*−/−*^:*Tmem106b*^−/−^ mice [35]. However, other lysosomal enzymes maintained elevated activity in *Grn*^*−/−*^:*Tmem106b*^−/−^ mice [35]. We obtained similar data, in which β-glucuronidase activity was suppressed in *Tmem106b*^*+/−*^ mice regardless of *Grn* genotype (Figs. 4, 5). Also, while complete Tmem106b knockout prevented open field, plus maze, and retinal abnormalities in *Grn*^*−/−*^ mice [35]; in both the prior study and ours, Tmem106b reduction failed to correct other key phenotypes, including lipofuscinosis and neuroinflammation in *Grn*^*−/−*^ mice [35] and social deficits in *Grn*^*+/−*^ mice (Fig. 2). Taken together, these data indicate that Tmem106b reduction may counteract some of the effects of progranulin insufficiency on lysosomal function, but does not completely rescue the lysosomal dysfunction induced by progranulin insufficiency (Fig. 6). Conversely, transgenic overexpression of TMEM106B in *Grn*^*−/−*^ mice worsens lipofuscinosis and lysosomal enlargement in *Grn*^*−/−*^ mice, which is consistent with the deleterious effects of TMEM106B overexpression on lysosomal function in cell culture [17, 20, 26–28, 34].

## Conclusions

While further study is needed, the data obtained from this study suggests that reduction of TMEM106B levels may not be the primary mechanism by which *TMEM106B* SNPs protect against FTD due to *GRN* mutations. Other aspects of these SNPs in linkage disequilibrium, such as the S/T coding variant, may alter TMEM106B function or interaction with other lysosomal proteins. It is also possible that human TMEM106B and progranulin interact in ways not modeled by mice, making human cell lines a potential model for further research. Such studies would complement the strength of mouse models in uncovering the molecular mechanisms that arise the aging brain. Given the clear protective effect of *TMEM106B* SNPs against multiple FTD subtypes and the emerging role of TMEM106B in aging and other neurodegenerative diseases [10–18, 49, 50], further study of TMEM106B’s effects in the brain are an important area for future research.

## Additional file

Additional file 1: **Figure S1.** Measurement of Glycosylated HexA and GCase by Western Blot. The western blots in fig. 3 measured the glycosylated forms of HexA and GCase, which was confirmed by loss of these bands after digestion with the glycosidase PNGase F. The glycosylated forms of HexA and GCase are labeled with black arrows, the unglycosylated forms with gray arrows, and nonspecific bands with asterisks. (DOCX 195 kb)

## Abbreviations

FTD: frontotemporal dementia
GCase: β-glucocerebrosidase
*GRN*: progranulin
Gusb: β-glucuronidase
HexA: β-hexosaminidase A
SNP: single nucleotide polymorphism
