# Supplementary material for: Partial Tmem106b reduction does not correct abnormalities due to progranulin haploinsufficiency
Source: Mol Neurodegener. 2018 Jun 22;13:32. doi: 10.1186/s13024-018-0264-6 (PMC6013889; doi:10.1186/s13024-018-0264-6)
Supplement: Supplementary file 1 — Figure S1. Measurement of Glycosylated HexA and GCase by Western Blot. The western blots in fig. 3 measured the glycosylated forms of HexA and GCase, which was confirmed by loss of these bands after digestion with the glycosidase PNGase F. The glycosylated forms of HexA and GCase are labeled with black arrows, the unglycosylated forms with gray arrows, and nonspecific bands with asterisks. (DOCX 195 kb) [file 13024_2018_264_MOESM1_ESM.docx]

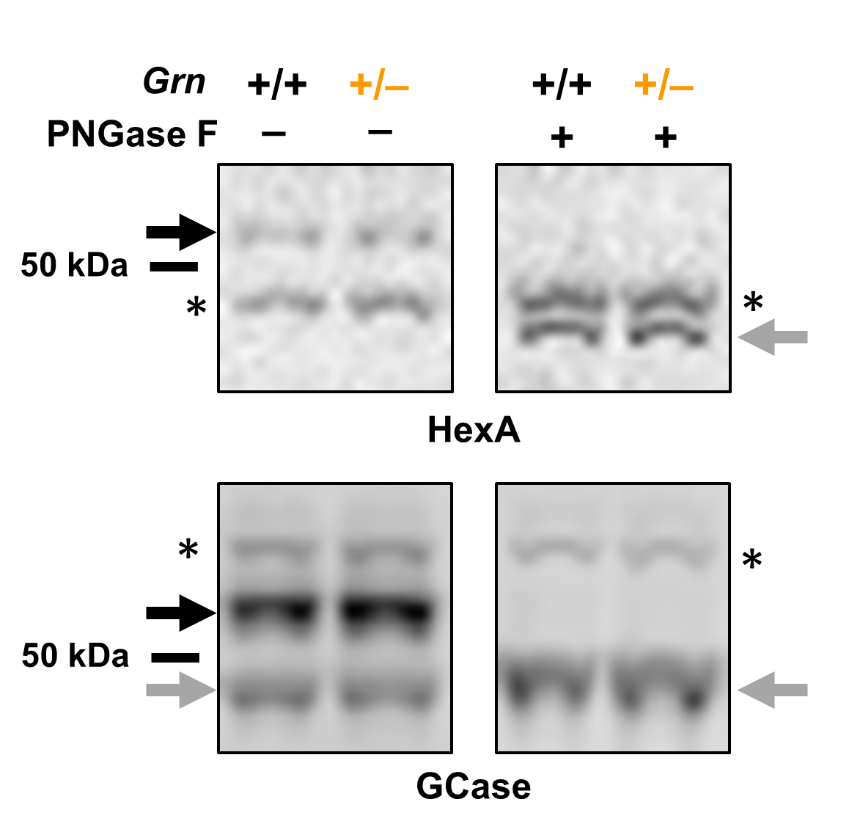


**Figure S1 – Measurement of Glycosylated HexA and GCase by Western Blot.**

The western blots in figure 3 measured the glycosylated forms of HexA and GCase, which was confirmed by loss of these bands after digestion with the glycosidase PNGase F. The glycosylated forms of HexA and GCase are labeled with black arrows, the unglycosidated forms with gray arrows, and nonspecific bands with asterisks.
